# The environmental impact of intravenous antimicrobial therapies: a comparison of OPAT and in-patient administration care pathways-right-to-reply

**DOI:** 10.1093/jacamr/dlaf193

**Published:** 2025-12-04

**Authors:** A Cole, J Aspin, S Laird, F Acri, S Galley, M Collins

**Affiliations:** Government Affairs and Market Access, Baxter Healthcare Ltd, Reading, UK; EHS and Sustainability, Baxter Healthcare Ltd, Reading, UK; Microbiology University Hospitals Coventry and Warwickshire (UHCW) NHS Trust, Coventry, UK; Marketing, Baxter Healthcare Ltd, Reading, UK; Consulting Environmental Resources Management (ERM), London, UK; Consulting Environmental Resources Management (ERM), London, UK

We would like to thank the authors for their comments and would like to take this opportunity to highlight that the ‘Care pathways: guidance for appraising sustainability’ document describes the application of LCA to treatment pathways, with the key objective being to enable more consistent quantification of the environmental sustainability of differing approaches to patient treatment, to give care providers a tool, and data to make decisions with environmental sustainability in mind. The guidance builds upon the requirements of the ISO14040 and ISO14044 Standards for Life Cycle Assessment, the Greenhouse Gas Protocol Product Life Cycle Accounting and Reporting Standard (Product Standard) and the Greenhouse Gas Accounting Sector Guidance for Pharmaceutical Products and Medical Devices and is intended to be used alongside these documents (page 11, guidance main document). The guidance requires primary data for the activities under the control of the organization undertaking the study and allows for secondary data to be used for the remainder of the care pathway where primary data collection is not practicable. The primary data requirements have been met by the organization undertaking the study and include the assessment of the elastomeric pump.

The secondary data, as provided by the guidance to facilitate care pathway assessments, does not include medicines. These are care pathway/condition specific and should be added on separately. This has been done within the assessment. Within all LCAs, there will be inconsistencies when relying on secondary data. The authors have clearly stated these in the discussion section of the paper.

It is necessary to interpret the results of a complex LCA or care pathway assessment in the light of data limitations. The limitations of the secondary data used were not found to be material (a key concept within the guidance) or sufficient to undermine the key finding that using outpatient parenteral antimicrobial therapy reduces the environmental impact of delivering antimicrobial therapy when compared to inpatient treatment.

## Funding

None to declare

## Transparency declarations

A.C., J.A. and F.A. are employees of Baxter Healthcare Ltd.

